# A geostatistical imputation of first floor elevation data for mapping flood vulnerability

**DOI:** 10.1007/s11069-026-08095-9

**Published:** 2026-04-06

**Authors:** Prarthana Raja, Yitong Li, Jie Gong

**Affiliations:** 1 Department of Civil and Environmental Engineering, Rutgers University, Piscataway, NJ 08854 USA; 2 Department of Civil and Environmental Engineering, University of Alberta, Edmonton, AB T6G 1H9 Canada

**Keywords:** First floor elevation (FFE), Geostatistical imputation, Kriging, Flood risk assessment, Semivariogram

## Abstract

First Floor Elevation (FFE) is a crucial indicator used in the United States for assessing the vulnerability of buildings to flood events. However, most buildings within floodplains lack accurate FFE data. On-site FFE data collection could be costly and time-consuming. To facilitate efficient FFE data collection, this paper explores the extent to which FFEs for an entire community’s building stock can be estimated using limited FFE data records. More specifically, this study leverages geostatistical imputation techniques to fill in missing FFE data. The proposed approach first stratifies buildings based on attributes such as foundation type and then applies Kriging to estimate missing FFEs by leveraging spatial relationships and distances between known data points. To demonstrate the applicability and validity, the approach is tested in three New Jersey townships: the inland township Manville and the two coastal townships Longport and Ventnor City. By transforming data into First Floor Height and stratifying by building type, the methodology achieved a Root Mean Square Error (RMSE) as low as 0.9893 ft in Manville, with variations in coastal towns due to unique structural and geographic factors. The findings highlight the potential of combining geostatistical modeling with building-specific attributes to enhance flood vulnerability assessments. This approach not only addresses critical data deficiencies but also supports informed decision-making in resilience planning and natural hazard mitigation.

## Introduction

Floods are the costliest and most frequently occurring natural disaster worldwide. Ensuring the safety of communities in flood-prone areas requires accurate assessments of building vulnerability. A critical factor in assessing a building’s vulnerability to flooding is the First Floor Elevation (FFE), defined by FEMA (Federal Emergency Management Agency) as the elevation of the top of the lowest finished floor in a building. When floodwater reaches the FFE, buildings experience significantly more damage, making it a key parameter in flood risk assessment and post-flood decision-making (Josephs et al. 2024). The primary source for obtaining FFE data in high-risk areas is the Elevation Certificate (EC), developed by FEMA. This certificate estimates a building’s risk by comparing its lowest floor elevation with the anticipated floodwater height during an event. However, obtaining an Elevation Certificate can be costly, with average prices around $600 and ranging from $170 to over $2,000, depending on property type and location (HomeAdvisor, 2022). Consequently, not all homes in flood zones have an Elevation Certificate, creating data gaps that complicate accurate flood vulnerability assessments. To address these data limitations, FEMA introduced Risk Rating 2.0, a policy effective April 1, 2023, aimed at modernizing the National Flood Insurance Program (NFIP) by shifting towards a fairer rating system (FEMA, 2022). Unlike traditional flood zones, Risk Rating 2.0 incorporates multiple variables such as FFE, ground elevation at the building site, foundation type, prior NFIP claims history, and distance from flooding source to assess the flood risk for each property. Although efforts have improved FFE data accuracy, achieving full coverage remains time-consuming, underscoring the need for reliable, scalable estimation methods in communities lacking Elevation Certificates.

A few research studies have focused on the estimation of First Floor Elevation (FFE). The methods used are broadly categorized as statistical-based approaches and image-based approaches. Statistical models make FFE predictions through learning the relationship between building attributes, such as foundation type, year built, and the observed FFE data. However, these methods are often constrained by sparse and incomplete training datasets because building-level elevation and infrastructure attributes are costly and time-intensive to collect (Wang et al. 2024). Image-based approaches employ computer vision and machine learning techniques to analyze data from sources like Google Street View (GSV) and LiDAR (Light Detection and Ranging) (Gao et al. 2023; Sorboni et al. 2024; Needham and McIntyre 2018; Xia and Gong 2021). These methods rely heavily on the quality and resolution of visual data, which can vary with the availability of high-quality aerial or street-view imagery. Their accuracy is often hindered by obstructions like trees or fences, privacy-related blurring, and outdated or incomplete imagery. Limited access to properties in private or restricted areas further constrains coverage. These challenges highlight the need for reliable FFE predictions when direct measurements are limited or incomplete.

Geostatistical models offer a promising solution to address these gaps. A geostatistical model is a statistical framework used to analyze and make predictions about spatially correlated data. These models account for the fact that observations collected at locations close to each other in space are often more similar than those collected at distant locations (Waters 2017). This concept applies to FFE estimation because buildings near each other are likely to have similar years of construction and follow the same building codes. The application of geostatistical models for post-disaster recovery has gained popularity in recent years. Notable applications include the prediction of building insurance claim counts and values impacted by hailstorms (Miralles et al. 2023) and the estimation of building damage ratings following earthquakes (Loos et al. 2020) and the prediction of highway flood damage following hurricanes (Li et al. 2023a, b, 2024; Chen et al., 2024). These studies illustrate the power of geostatistical models to predict variables of interest given sparse data, making them well-suitable for FFE prediction.

The primary goal of this research is to design a systematic approach to predict FFE given limited FFE data. The study leverages geostatistical imputation techniques to fill missing FFE data. The proposed approach involves two key steps: (1) stratifying buildings based on attributes such as foundation type, and (2) using spatial interpolation methods, particularly Kriging, to estimate missing FFEs by leveraging spatial relationships and distances between known data points. The research was implemented in one inland and two coastal New Jersey townships, Manville, Longport, and Ventnor City, each presenting unique geographical and structural characteristics. The results demonstrated that Kriging, especially when combined with stratification by building type, significantly improves prediction accuracy. These findings underscore the adaptability and precision of the proposed methodology, offering a reliable framework for estimating FFEs in diverse settings, particularly in regions where data availability is limited.

## Literature review

Predicting First Floor Elevation (FFE) is essential for assessing flood risk and enhancing coastal resilience. Over time, various methodologies have been developed to estimate FFE, utilizing both statistical and image-based processing techniques. This review synthesizes recent research on these methodologies, highlighting key advancements and identifying their limitations.

### Statistical and machine learning-based appproaches

Statistical and machine learning-based approaches estimate First Floor Elevation (FFE) by leveraging building details like foundation type, construction year, and flood zone, along with geospatial data such as DEM (Digital Elevation Model) and BFE (Base Flood Elevation). Methods such as Random Forest and GAM (Generalized Additive Model) have demonstrated good performance in modeling the relationships between building details and FFE. However, the applicability of these models is often limited by sparse datasets and regional variations. This highlights the importance of integrating more comprehensive and context-specific datasets to improve prediction accuracy and generalizability.

In Cannady et al. (2019), a Random Forest model was developed using elevation certificate data to predict First Floor Height (FFH) for the Hampton Roads region. The methodology involved using Random Forest, a machine learning algorithm known for handling complex, non-linear relationships alongside a suite of predictors, including foundation type, year built, and differences in grade. The model was trained and validated on a subset of elevation certificate data, with the foundation type emerging as the most influential variable. Despite its success in identifying key predictors, the study faced limitations due to the sparse and homogeneous nature of the dataset, which reduced its generalizability to other regions or diverse building types. Additionally, discrepancies in how attributes were recorded across localities added further complexity to model scalability. Similarly, Taghinezhad et al. (2020) explored statistical imputation methods for estimating FFE in flood-mitigated single-family homes in Louisiana. This study compared multiple techniques, including Random Forest, Generalized Additive Models (GAM), and multiple linear regression. GAM, a flexible regression technique that handles non-linear relationships, demonstrated the highest accuracy in imputing missing FFE values. The model incorporated key building attributes such as foundation type, BFE, and DEM. To evaluate model performance, the study employed cross-validation methods like Leave-One-Out Cross-Validation (LOOCV) to assess prediction error. While the GAM model excelled in handling complex relationships, the study’s reliance on an incomplete distribution of foundation types in the dataset posed significant challenges. This creates challenges for applying the model across diverse structural types and geographical contexts.

Among these studies, building attributes have been demonstrated to provide critical insights into flood vulnerability. Cannady et al. (2019) highlighted that buildings with elevated foundations are less prone to flood damage, emphasizing the importance of foundation type and height. Chen et al. (2022) advanced this field by integrating image-based techniques, using object detection algorithms to extract key building features from visual data, thereby enhancing FFE estimations. Standardized classification systems like FEMA’s Building Diagram Classification are pivotal in these methodologies. FEMA’s diagrams categorize structures by foundation type and elevation, providing a consistent framework for FFE estimation and flood risk assessments. For instance, slab-on-grade buildings are more vulnerable compared to pier-and-beam structures. Such classifications also guide floodplain management and insurance evaluations (FEMA 2023). By refining building classifications and leveraging advanced statistics, these methodologies enhance the accuracy and applicability of FFE estimation, enabling more targeted flood mitigation strategies and resilience planning. However, the need for diverse and comprehensive datasets remains critical for improving model reliability and extending their generalizability across varied geographic and structural contexts.

### Image-based processing approaches

In recent years, image-based processing techniques have gained attention as a powerful alternative to traditional statistical methods. These approaches leverage advancements in computer vision and machine learning to analyze visual data, such as images from Google Street View (GSV), LiDAR scans, and aerial imagery from drones. Image-based methods for FFE estimation can be broadly categorized into two types: those that utilize street-level data and those that rely on aerial data.

Gao et al. (2023) employed an object detection model based on YOLO-v5 (You Only Look Once-v5) to detect the location of doors in Google Street View (GSV) images. The FFE was then calculated based on the predefined general door height and the door bottom elevation. However, the use of GSV data presents several limitations. In particular, outdated images may not accurately reflect the current state of buildings, especially if renovations or other changes have occurred. Additionally, the dataset used in this study was limited in terms of the diversity of building types and designs, which affects the generalizability of the results. Buildings located along alleys or in areas not visible from the street posed further challenges, as the GSV privacy feature allows users to blur their homes. While this feature enhances privacy, it restricts data availability to key structural features needed for flood risk assessments. Together, these limitations underscore the challenges in relying solely on street-level imagery for comprehensive flood vulnerability studies. Building on this work, Sorboni et al. (2024) expanded the use of the YOLOv5s algorithm to GSV images to detect a wider range of building elements, including front doors, stairs, and the overall building extent. This study introduced a novel application to identify basement windows and assess the presence of basement critical factors in flood risk assessment. While this represented significant progress, the study highlighted persistent challenges, particularly obstructions like trees, shrubs, or fences in GSV images that hinder accurate measurements.

To address these limitations, Xia and Gong (2024) demonstrated the use of street-level LiDAR data, which showed significant improvements in the accuracy of FFE estimates compared to GSV images. LiDAR data, with its ability to capture 3D spatial details, overcomes some of the challenges posed by GSV imagery, such as obstructions and outdated visuals. However, LiDAR data acquisition is not immune to limitations, as obstructions like dense vegetation or man-made structures can still interfere with measurements. Additionally, the cost and logistical challenges of acquiring LiDAR data make its widespread application difficult. Chen et al. (2022) presented another innovative approach that used GSV images to estimate multiple building attributes concurrently, including foundation height, foundation type, building type, and the number of stories. This approach combined image classification and object detection techniques to classify images and extract specific building characteristics. The estimation of foundation type and height was treated as a regression problem, solved using Multitask Learning and Convolutional Neural Networks (CNNs). However, a significant limitation of this method was the quality of the GSV images. Many images were unclear or incomplete, obstructed by objects like fences, trees, or vehicles, or depicted buildings that were too small for effective analysis. These limitations created challenges in accurately identifying and estimating the building attributes necessary for reliable flood risk assessment.

Aerial imagery, captured by Unmanned Aerial Systems (UAS), has also emerged as a valuable tool for FFE estimation. In their studies, Diaz et al. (2022) and Diaz et al. (2024) utilized UAS to estimate FFE by capturing Structure-from-Motion (SfM) imagery and creating accurate 3D models of selected residential communities in Galveston, Texas. High-resolution aerial imagery and photogrammetric modeling were crucial for obtaining precise FFEs. The FFEs were estimated using Lowest Service Grade (LSG), Lowest Adjacent Grade (LAG), and Highest Adjacent Grade (HAG) and were found to be statistically comparable to Elevation Certificate (EC) measures. The 2024 study further refined this method by employing high-precision drone-photogrammetry, supplemented with building inventory and geoinformation data from online sources, to accurately map the flood vulnerability of individual structures. However, both studies noted significant limitations, such as tree coverage obstructing the view of the building elevation. This makes it challenging to estimate the elevation data when the view is unclear. Another innovative approach was explored by Kakade et al. (Kakade et al., n.d.), who conducted a pilot study using drones equipped with thermal imaging to estimate the Lowest Floor Elevation (LFE). The study calculated LFE by detecting color changes between the building and foundation structures due to temperature differences. While this approach showed promise, it was limited to only two buildings. Further research is needed to validate the methodology on a larger scale.

In summary, predicting FFE remains a complex and multifaceted challenge, despite significant advancements in both statistical-based and image-based methodologies. Statistical approaches often suffer from limitations related to data quality and diversity. These limitations hinder the generalizability of the models, making it difficult to apply the findings across different regions and building types. Image-based methods offer innovative solutions but face challenges in terms of data acquisition and obstructions. Aerial methods, such as those utilizing drones, have shown great promise in providing high-resolution data for FFE estimation. However, these methods are highly dependent on the quality and resolution of the visual data, which can vary significantly based on the availability of high-quality aerial or street-view imagery. Their reliability is also compromised by objects like trees, fences, or buildings that are not visible from the street. Privacy protections, such as image blurring on platforms like Google Street View, can also obscure critical features for accurate FFE estimation (e.g., windows, doors, and foundations). Additionally, the timeliness and resolution of images may not be timely or adequately captured in street-level imagery, leaving significant gaps in the dataset. These limitations underscore the need for a robust imputation technique to fill in the gaps where data collection is challenging.

### Geostatistical models and kriging in disaster management

Geospatial models are essential tools for understanding, predicting, and mitigating the effects of natural and human-induced hazards. By integrating diverse spatial datasets such as topography, hydrology, land use, and meteorology into comprehensive frameworks, detailed spatial analysis and visualization can be performed. Geospatial models have been widely applied for disaster management, urban planning, and environmental conservation, providing critical insights for informed decision-making. With advancements in geospatial technologies and the availability of high-resolution data from sources like satellite imagery and LiDAR, these models have become indispensable for disaster preparedness and mitigation (Akhyar Akhyar et al. 2024).

In disaster management, geospatial models are particularly effective for flood risk assessment and understanding socio-economic impacts. For example, Tariq et al. (2023) evaluated flash flood susceptibility in the Potohar region of Pakistan by integrating hydrogeomorphic factors such as slope, soil type, and rainfall patterns within a geospatial decision-support framework. Similarly, Gambo et al. (2024) investigated flood risk and multidimensional poverty in Jigawa, Nigeria, highlighting spatial associations between high-risk zones and socio-economic vulnerability. While these studies emphasize integrated spatial assessment for flood risk mapping, recent work has increasingly focused on geostatistical models that explicitly account for spatial dependence and uncertainty. In this context, advanced geostatistical approaches such as the Bayesian Generalized Linear Geostatistical Model (BGLGM) have been used to predict highway inundation during flood events and to address data sparsity challenges (Li et al. 2023a, b, 2024). Application of this model during Hurricane Harvey in Harris County, Texas demonstrated strong predictive performance for assessing flood impacts on transportation networks.

Kriging is a type of geostatistical model that makes spatial predictions by leveraging spatial correlations between observations. For instance, Di Maio et al. (2022) applied Parallel Density Scanned Adaptive Kriging to enhance Tsunami hazard assessments. This method has been demonstrated to reduce computational demands while improving hazard estimations for coastal infrastructure. Similarly, Ali and Rahman (2022) introduced a kriging-based regional flood frequency analysis for South-East Australia. Their technique used data from 558 catchments to predict flood quantiles. Loos et al. (Loos et al. 2020) have developed a geospatial data integration framework to rapidly assess post-earthquake damage to buildings. This framework employs regression kriging, which combines regression analysis with kriging interpolation, to predict earthquake-induced building damage ratings. The results demonstrated improved prediction performance with a small training data size.

Kriging is a promising approach to address limitations in estimating First Floor Elevation (FFE) faced by traditional statistical and image-based approaches. While statistical methods rely on complete datasets and image-based techniques depend on the quality of available imagery, kriging interpolates FFE values using spatial correlations. This capability enables accurate assessments in areas with limited or obstructed data, making kriging a valuable tool for flood risk assessments, disaster planning, and targeted mitigation strategies, particularly in underrepresented regions.

## Methodology

**Fig. 1:**
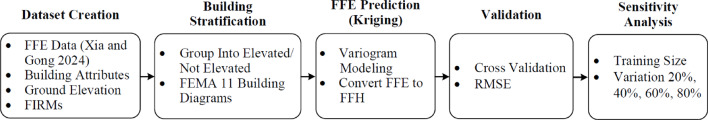
Methodology

The methodological framework developed in this study is designed to address gaps in First-Floor Elevation (FFE) data by integrating existing datasets with geostatistical modeling. The workflow consists of five sequential steps (Fig. 1). First, a dataset of building-level FFE values was assembled by combining the LiDAR-based extraction dataset of Xia and Gong (2024) with building attributes, ground elevation, and FEMA Flood Insurance Rate Maps (FIRMs). Second, buildings were classified as either elevated or not elevated and subsequently stratified according to FEMA’s 11 standard building diagrams. Third, kriging was employed to estimate missing FFE values by modeling spatial autocorrelation and removing elevation trends through the conversion of FFE to First-Floor Height (FFH). Fourth, the accuracy of predictions was evaluated through cross-validation, comparing observed and predicted FFEs against error thresholds. Finally, a sensitivity analysis was conducted to assess the robustness of the methodology under varying sample sizes of observed data. Together, these steps provide a systematic approach for estimating FFE across diverse urban and coastal environments where direct measurements are limited or incomplete.

### Dataset creation

Two types of information were collected and processed for FFE prediction: (1) building design information (e.g., location, number of stories, elevation status) and (2) building FFE values.

Building design information was collected through a parcel-by-parcel virtual survey that was conducted in February 2023 using Google Street View. Surveyors recorded whether each residence is elevated above grade, contains a basement, or is configured as a split-level, and assigned the appropriate FEMA Building Diagram category. Observations were entered in real time via a Google Sheets form (see attributes in Table 1). The completed spreadsheet was subsequently joined to the GIS parcel layer after editing municipal parcel shapefiles to reflect tax-parcel boundaries. Because multiple-deed lots often merge into a single ownership unit, it was necessary to establish a one-to-one alignment between field-verified building data, revised tax-parcel geometry, ground elevation, and FFE values. This alignment provides a consistent, georeferenced foundation for flood-risk analysis. Boundaries, tax-parcel polygons, road centerlines, and FEMA Flood Insurance Rate Maps (FIRMs) were downloaded from the New Jersey Bureau of GIS open-data portal. These vector layers form the spatial framework to which all other datasets are referenced, ensuring consistent geolocation across subsequent analyses.

FFE information for buildings was extracted using an automated method that trains a deep learning model on projected LiDAR images of building facades. The workflow begins by clipping the point cloud for each parcel and projecting it to a bird’s-eye view to isolate individual structures. Building façades are then detected and re-projected into a frontal view to create an intensity and elevation raster. Using the intensity raster, a YOLOv5 object-detection model was trained to identify key façade elements (e.g., windows, doors, and garage doors). Adjacent ground elevation is extracted from the same LiDAR dataset, and FFE is calculated as the vertical difference between ground level and the detected sill or threshold heights. A comprehensive description of the data-processing pipeline, model architecture, and validation is provided in Xia and Gong (2024). A summary of the processed data fields and their explanations is shown in Table 1.

**Table 1:** Summary of Data Sources

Name	Description	Source
Property address	Address of a building	NJ parcel data
Longitude	The longitude of a building	NJ Bureau of GIS
Latitude	The latitude of a building	NJ Bureau of GIS
Number of stories	Number of stories (1, 2, and 3)	Survey for GSV
Structure elevation	Is the structure raised above the adjacent ground or not	Survey for GSV
Basement	Does the structure have a basement	Survey for GSV
Split-level structure	Is the structure split or bi-level	Survey for GSV
First floor elevation	The vertical height of the finished surface of a building’s first occupied floor	LiDAR Data
Adjacent ground elevation	The elevation of the ground around the structure	LiDAR Data

### Building stratification

The Federal Emergency Management Agency (FEMA) is the primary agency that determines a building’s FFE through a detailed analysis of building diagrams following the Elevation Certificate (EC) instructions (FEMA 2023). FEMA’s EC instructions specify standardized building diagrams to ensure FFE measurements are properly referenced based on the building’s foundation type, enclosure configuration, and other structural attributes. Such standardization is essential because each foundation type responds differently to flood forces, which directly affect vulnerability, insurance premiums, and mitigation options. As a result, classification of building diagrams is an essential step to ensure accurate FFE predictions. In addition, a building’s elevation status is a critical factor because elevated structures, such as those on piers or crawlspaces, typically have higher FFEs compared to non-elevated buildings.

The first step of the methodology is to assign every structure to one of FEMA’s 11 building-diagram categories. These eleven diagrams fall into two broad groups: non-elevated buildings with foundations resting at or below grade (e.g., slab-on-grade, full basements) and elevated buildings with the lowest occupied floor raised above grade (e.g., crawlspaces, piers, piles, or stilts). The table below (Table 2) presents the 11 FEMA building diagrams, grouped into the two elevation classes described above. Each of these diagrams illustrates where the FFE (indicated as a red line) is taken.

**Table 2:** FEMA Building Diagrams and their explanation

Elevated Status	Building Diagram Number	Building Diagram Illustration with FFE	Explanation
Not Elevated	1A		Slab-on-grade with no basement, with or without attached garage.
	1B		All raised-slab-on-grade or slab-on-stem-wall with fill. With or without attached garage.
	2 A		All buildings (other than split-level) with a basement, detached or row type, with or without an attached garage.
	2B		All buildings with a basement (other than split-level) and all high-rise buildings with basements—detached or row-type, with or without an attached garage—whose doors and other means of egress are below ground level on all sides.
	3		All Split-level buildings are slab-on-grade, detached or row type, with or without an attached garage.
	4		All Split-level (other than slab-on-grade) detached or row type, with or without attached garage.
Elevated	5		All buildings are elevated on piers, post piles, columns, or parallel shear walls. No obstruction below the elevated floor,
	6		All buildings are elevated on piers, post piles, columns, or parallel shear walls with full or partial enclosure below the elevated floor.
	7		All buildings are elevated on a full-story foundation wall with a partially or fully enclosed area below the elevated floor.
	8		All buildings elevated on a crawlspace with the floor of the crawlspace at or above grade on at least one side, with or without an attached garage.
	9		All buildings (other than split-level) elevated on a subgrade crawlspace with or without an attached garage.

### Prediction of FFE using kriging

Once the buildings are classified by building diagrams, the second step of the methodology is to apply Kriging to estimate buildings with unknown FFEs. This methodology relies on Tobler’s first law of geography: “everything is related to everything else, but near things are more related than distant things” (Tobler 1970; Waters 2017). The first step of Kriging is to generate a variogram to quantify the spatial autocorrelation of FFEs among buildings at different locations. The variogram plots semivariance on the Y-axis, representing the extent of variability between FFEs of different buildings, against lag distance on the X-axis, indicating the geographic separation between data points (Shekhar et al. 2018). The semivariance is calculated using Eq. 1.

1 $$ \begin{array}{*{20}c} {\gamma \left( h \right) = \frac{1}{{2N}}~\mathop \sum \limits_{{\left\{ {h_{{\left\{ {ij} \right\}}} \approx h} \right\}}}^{~} ~\left( {\emptyset _{i} - \emptyset _{j} } \right)^{2} ~~} \\ \end{array} $$

Where$\gamma\left(h\right)$ represents the semivariance for a given lag distance *h*, which quantifies the average squared differences of the First Floor Elevation (FFE) values ($\varnothing$) separated by a distance *h*. The is the squared difference between the FFE values at two building locations *i* and *j*. This measures the dissimilarity in FFE values for buildings separated by a distance *h*. The $\frac{1}{2N}$ is a constant factor ensuring the semivariance is calculated as half the mean of squared differences, where $N$ is the number of data points for a paired building.

**Fig. 2:**
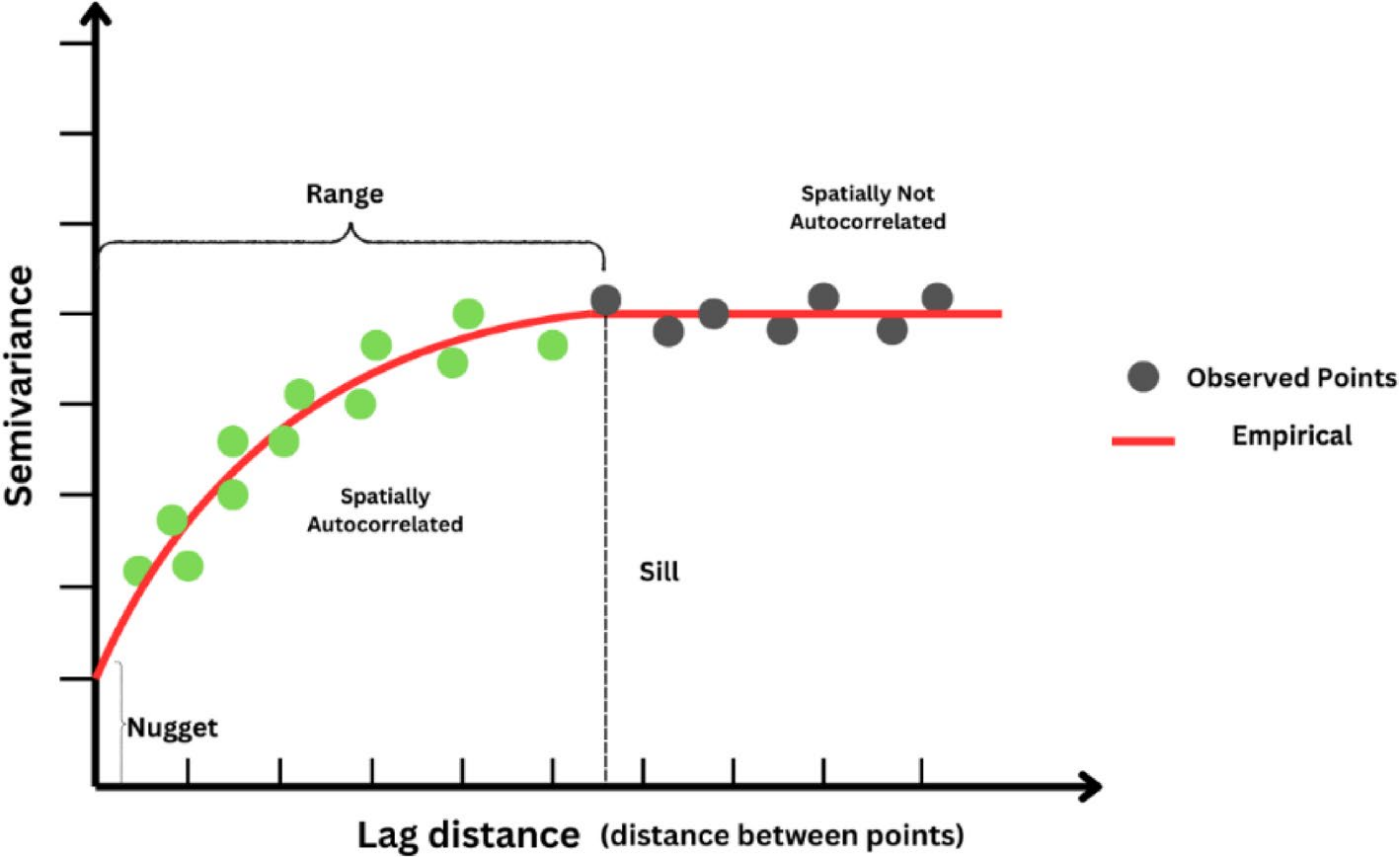
Key components of a Variogram

Based on these data, the empirical variogram can be generated by plotting the computed semivariances against the corresponding distances (shown in Fig. 2). The parameters range, sill, and nugget define the spatial dependency of the variogram. Range defines the maximum distance within which spatial autocorrelation of FFE values is observed. Beyond this range, FFE values are treated as spatially independent. Sill represents the semivariance level where spatial correlation stabilizes, indicating that FFE values separated by this distance no longer exhibit meaningful correlation. Nugget accounts for minor errors in FFE measurements or inherent variability in building characteristics at a fine spatial scale.

Ordinary Kriging is selected to predict First Floor Elevations (FFEs) for buildings at unobserved locations. Stationarity is also an important property for kriging, as kriging works best when the data’s statistical properties, such as mean and variance, remain constant. FFE measures the height of the first finished floor relative to a fixed reference point, typically sea level, which can be influenced by variations in ground elevation. These variations introduce trends into the data, which can compromise the accuracy of kriging. To remove these trends and achieve stationarity, the First Floor Elevation is replaced with First Floor Height (FFH). First Floor Height measures the height of the first finished floor relative to the ground on which the building is situated. By focusing exclusively on First Floor Height, the influence of ground elevation is eliminated, effectively removing the trend and allowing for a more accurate and stationary dataset, which is ideal for kriging.

FFH is calculated following Eq. 2.

2 $$ \begin{array}{*{20}c} {FFH\left( s \right) = FFE\left( s \right) - GND\left( s \right),} \\ \end{array} $$

Where $GND\left(s\right)$ denotes ground elevation at the building location $s$. To capture how FFH varies with distance, the experimental variogram is computed following Eq. 3.

3 $$ \begin{array}{*{20}c} {\gamma \left( h \right) = \frac{1}{{2N\left( h \right)}}~\mathop \sum \limits_{{\left\{ {\left( {i,j} \right):s_{i} - s_{j} \approx h} \right\}}} \begin{array}{*{20}c} {\left[ {FFH\left( {s_{i} } \right) - FFH\left( {s_{j} } \right)} \right]^{2} ~~} \\ \end{array} } \\ \end{array} $$

Then, the variogram function (e.g., Matérn, Spherical, and Exponential models) that best describes spatial dependence is selected to fit an empirical variogram. From the fitted empirical variogram, the parameters nugget, range, and sill are estimated. These parameters define the spatial covariance structure. Ordinary kriging applies to this covariance structure to compute a system of linear Equations that assigns weights ${\lambda}_{i}$to the $n$ nearest buildings with known FFH surrounding a building with unknown FFH at the location ${s}_{0}$. The objective is to produce the best linear unbiased estimate of FFH at ${s}_{0}$. The kriging estimator is expressed as:

4 $$ \begin{array}{*{20}c} {\widehat{{FFH}}\left( {s_{0} } \right) = \sum _{{i = 1}}^{n} \lambda _{i} FFH\left( {s_{i} } \right),\sum _{{i = 1}}^{n} \lambda _{i} = 1} \\ \end{array} $$

Where $\widehat{FFH}\left({s}_{0}\right)$ represents the estimated FFH of a building at the location ${s}_{i}$ and ${\lambda}_{i}$ The weights are determined from the kriging Equations based on the variogram model (Oliver and Webster 1990). In the final step, the predicted First Floor Height (FFH) at location s₀ is converted back to First Floor Elevation (FFE) by adding it to the ground elevation at ${s}_{0}$, consistent with Eq. 5.

5 $$ \begin{array}{*{20}c} {\widehat{{FFE}}\left( {s_{0} } \right) = \widehat{{FFH}}\left( {s_{0} } \right) + GND\left( {s_{0} } \right)~} \\ \end{array} $$

### Model validation

To evaluate the accuracy of predicted First Floor Elevations (FFEs), cross-validation was conducted by partitioning the dataset into training and validation subsets. In each validation run, 20% of the observed FFE values were randomly selected as training data, and the remaining 80% of buildings were withheld for prediction. Model performance was assessed by comparing predicted and observed FFEs using Root Mean Square Error (RMSE) and mean error as evaluation metrics. To ensure robustness against sampling variability, this procedure was repeated five times using independent random subsamples. RMSE values were computed for each run and summarized using the mean RMSE and its variability (reported as standard deviation and range). This repeated subsampling approach was applied consistently across all study areas, including Manville, Longport, and Ventnor City.

RMSE thresholds were defined following commonly accepted FEMA and survey accuracy standards, where an error margin within 1 ft is considered accurate and values between 1 and 2 ft are considered acceptable. These thresholds are consistent with reported uncertainties in conventional elevation data products, as Elevation Certificates and associated survey measurements may exhibit errors exceeding 1 ft in a substantial portion of surveyed buildings, with typical survey control point and benchmark uncertainties ranging from approximately 0.5 to 1 ft and, in some cases, several feet (Maune 1997). All elevation values are reported in feet (ft).

### Sensitivity analysis

To test the robustness of the proposed methodology under varying levels of data availability, a sensitivity analysis was performed by systematically altering the proportion of observed FFE values used as input. Incremental percentages of observed data (e.g., 20%, 40%, 60%, 80%) were used as training datasets, with the remaining samples held out for validation. Changes in RMSE across different training sizes were recorded to evaluate how prediction accuracy responds to varying data densities. This analysis provides insights into the minimum data requirements necessary for reliable implementation of the methodology in communities with sparse building-level elevation data.

For each training-data proportion (20%, 40%, 60%, and 80%), the sensitivity analysis adopted the same repeated subsampling procedure used in model validation. Specifically, five independent random subsamples were generated for each proportion, and RMSE was computed for each run. The reported RMSE values represent the mean RMSE across the five subsamples, allowing the stability of model performance to be evaluated under varying data availability.

## Case study results

Three towns in New Jersey, Manville, Longport, and Ventnor City, were selected to demonstrate the applicability and validity of the proposed methodology. The following sections first describe the study areas and then apply the methodology to the inland and coastal towns, respectively.

### Study area

Each of the selected towns was introduced to highlight its unique geographic and structural characteristics that shape flood vulnerability.

Manville is a borough in Somerset County, located in the Raritan Valley region and part of the New York metropolitan area. Manville covers a total area of 2.45 square miles, of which 2.36 square miles is land and 0.09 square miles is water (3.59%) (U.S. Census Bureau 2021). The borough is mostly situated in a low-lying floodplain surrounded by rivers and streams, including the Raritan River to the north, the Millstone River to the east, and Royce Brook to the south. Recurrent flooding has been observed near the Raritan and Millstone Rivers. Approximately 250 residential structures are located within the 1% annual chance floodplain in Manville. The primary cause of flooding in Manville is fluvial flooding from the Raritan and Millstone Rivers, which occurs when river flow exceeds capacity, causing water to breach the banks and flood surrounding areas (US Army Corps of Engineers 2016).

**Fig. 3:**
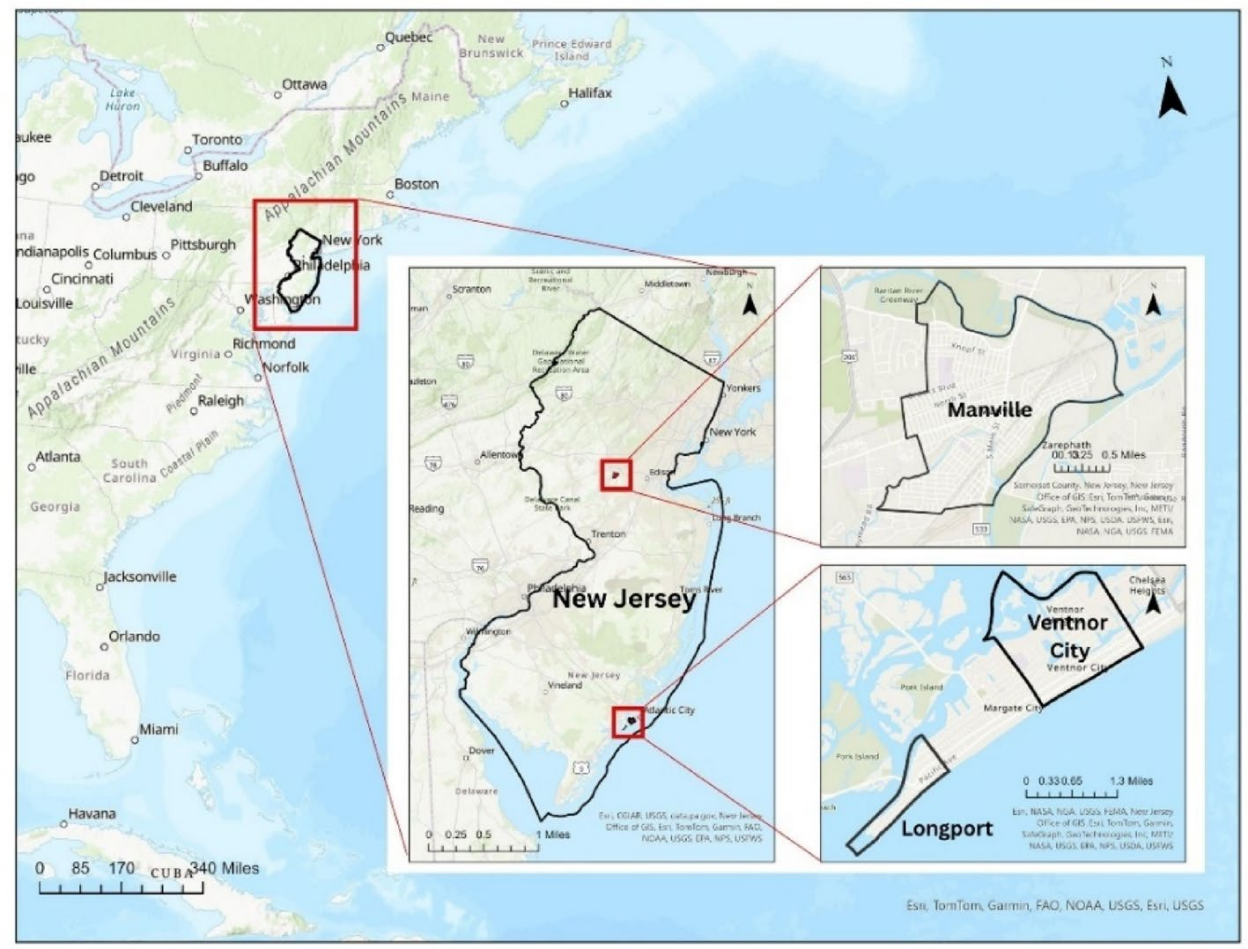
Map showing Study Areas, Manville, Ventnor City, and Longport in New Jersey

Longport is a small shore borough at the southernmost tip of Absecon Island in Atlantic County, as shown in Fig. 3. According to the U.S. Census Bureau’s 2020 Gazetteer Files, the borough has a total area of 1.56 square miles (4.03 km²), including 0.40 square miles (1.02 km²) of land and 1.16 square miles (3.01 km²) of water—about 74.5% water (U.S. Census Bureau 2021). Longport is surrounded by water on three sides, with the bay on the northwestern side and the ocean on the southeastern side, making it vulnerable to flooding. As a coastal town, flooding can be caused by hurricanes, tropical storms, coastal storms, and occasional high tides with heavy rain events. Flooding in Longport can cause water to fill streets and low-lying areas. Depending on ground elevations, the depth of the water can range from six inches to two feet. Areas with lower elevation are more likely to experience deeper flooding. This kind of flooding happened in the March 1962 storm and happened again in the December 1992 storm. During both events, the entire town of Longport was covered by floodwater (“Flood Information - Borough of Longport, Atlantic County, New Jersey” 2024).

Ventnor City is a coastal town located on Absecon Island in Atlantic County, New Jersey. The town is bordered by Atlantic City to the northeast and Margate City to the southwest, as shown in Fig. 3. According to the U.S. Census Bureau’s Gazetteer, Ventnor City covers a total area of 3.52 square miles (9.13 km²), of which 1.96 square miles (5.07 km²) is land and 1.57 square miles (4.06 km²), or 44.52%, is water (U.S. Census Bureau 2021).Ventnor City faces significant flood risks as its location is bordered by the Atlantic Ocean to the southeast and numerous inland waterways. Like neighboring coastal communities, the town is vulnerable to storm surges and tidal flooding. Due to its low-lying geography, parts of Ventnor City are within designated flood zones, particularly susceptible during hurricanes, tropical storms, and high-tide events. Notably, Ventnor has experienced substantial flooding in severe weather events, including the 1962 Ash Wednesday Storm and Superstorm Sandy in 2012, which caused widespread water inundation across low-lying areas and considerable damage to infrastructure (Coastal Coalition 2024).

### Inland town

#### Dataset creation and building stratification

**Fig. 4:**
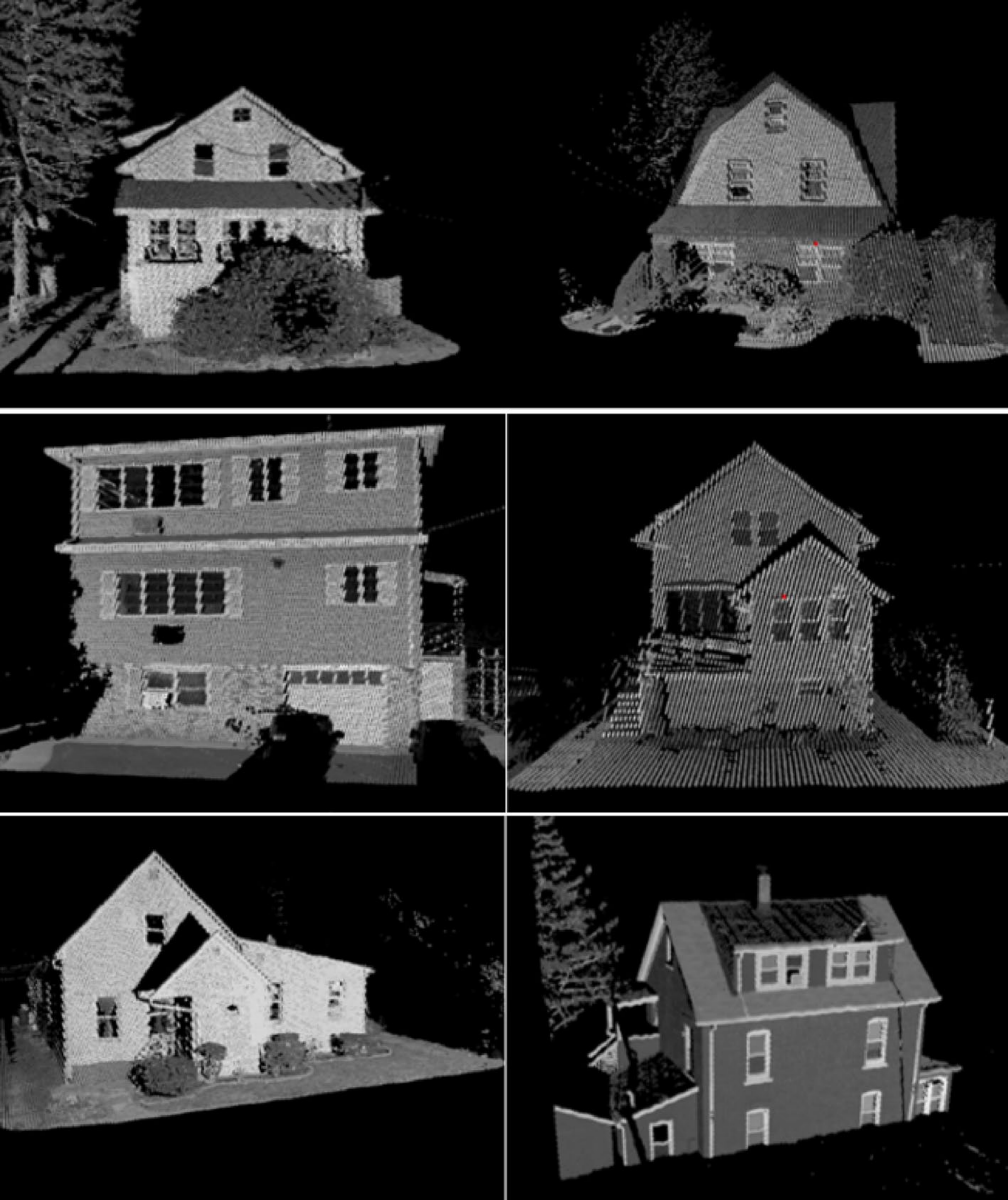
Examples of obstructed LiDAR views that limit FFE extraction

**Fig. 5:**
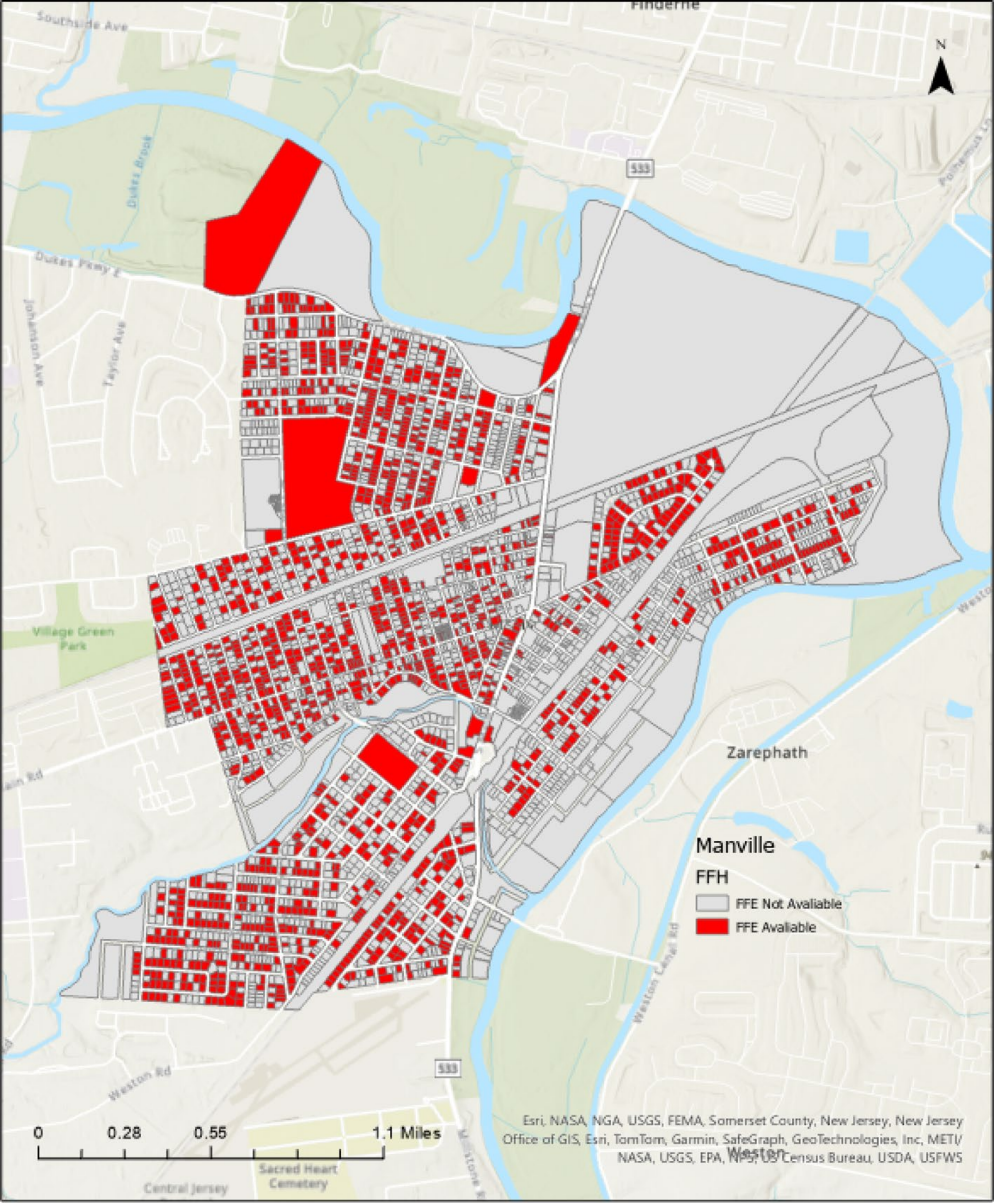
FFE availability by parcel in Manville

In Manville, FFE values were successfully generated for 1,950 out of 3,000 parcels during the dataset creation process (Xia and Gong 2024). The remaining 1,050 parcels were excluded because the front door was not visible in the point cloud (shown in Fig. 4), due to obstructions like trees, fences, or parked cars. The front door visibility was also limited by orientation issues, such as entrances not facing the street or unfavorable scanning angles. Figure 5 shows which parcels have available FFE data and which do not. Here, “data availability” refers specifically to the presence of FFE values, not to the existence of LiDAR data. It reflects whether the front door was sufficiently visible in the point cloud to allow for FFE extraction. These visibility-related limitations stem from the dataset creation process described in Xia and Gong (2024), not from the present study. As a result, the 1,950 parcels with valid FFE values were used for Manville analyses.

**Fig. 6:**
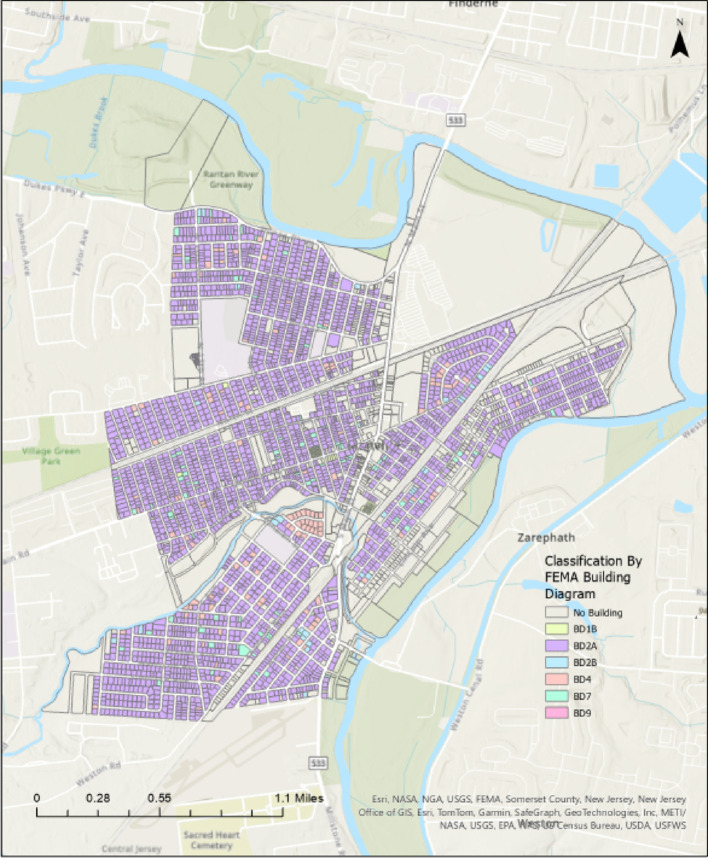
Map of Manville showing parcels classified by Building Diagram

When the dataset is grouped according to the FEMA building-diagram scheme, roughly 9% of the structures fall under Building Diagram 2 A. This diagram has non-elevated homes with fully below-grade basements and no exterior openings, as illustrated in Fig. 6. Given that the majority of homes in Manville fall under the 2 A building diagram, this building diagram was selected to demonstrate the applicability of ordinary kriging for FFE estimation.

#### FFE prediction and validation

**Fig. 7:**
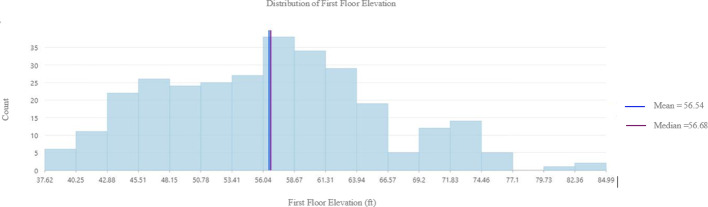
Histogram of First Floor Elevation (FFE) values (ft)

**Fig. 8:**
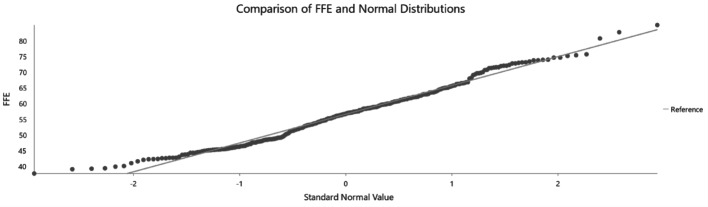
Quantile-Quantile (QQ) plot to show if data is normally distributed

For kriging to be effectively applied, the data must adhere to the assumptions of normality and stationarity. Therefore, the dataset will be examined to assess whether it meets these statistical requirements. The histogram, as shown in Fig. 7, and the Q-Q plot, as shown in Fig. 8, indicate that the data follows a normal distribution. This is evident from the bell-shaped curve of the histogram and the close alignment of the mean and median values. Additionally, the Q-Q plot supports normality, as the data points closely follow the reference line.

**Fig. 9:**
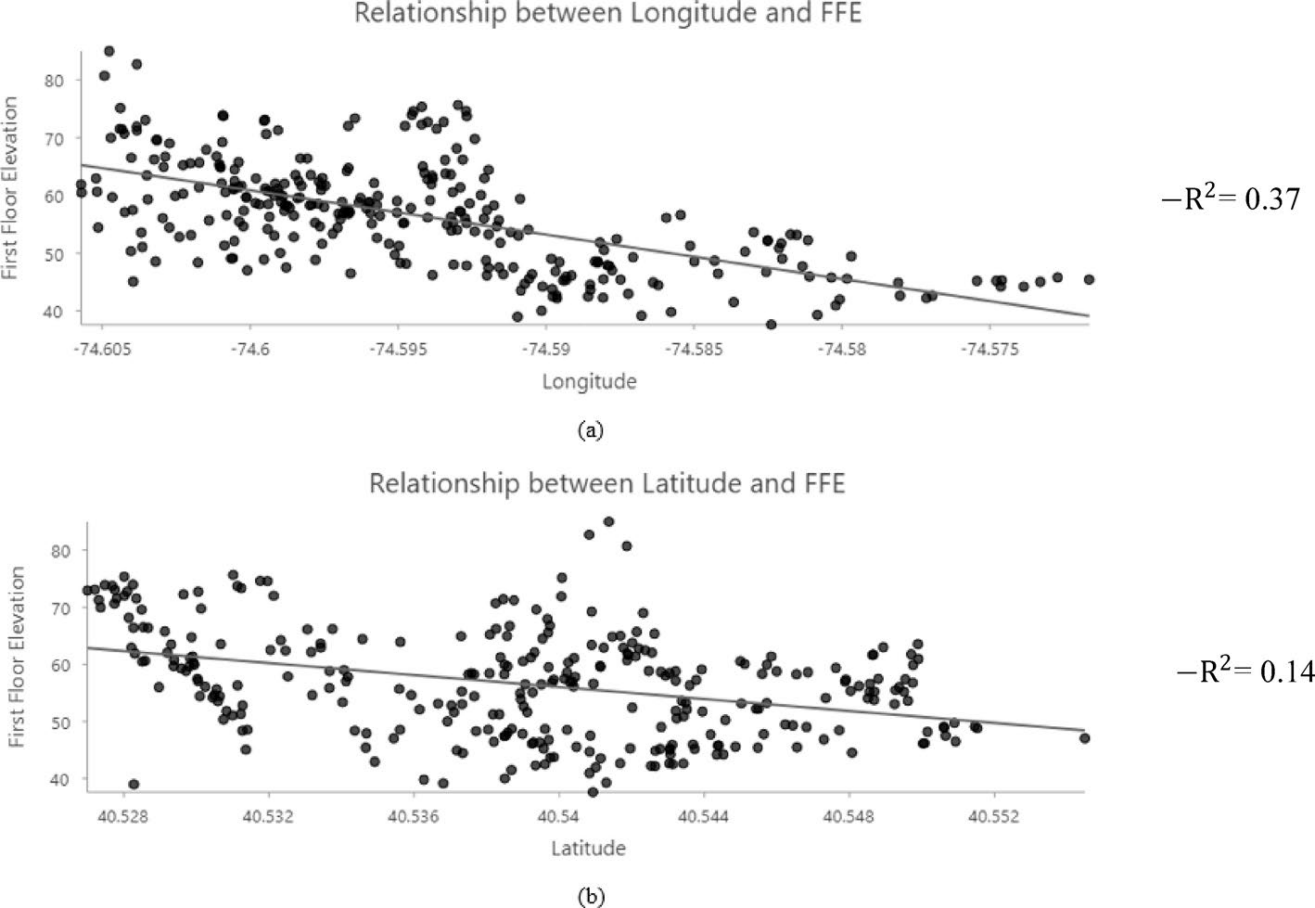
Relationship between First Floor Elevation (FFE) and geographic location: (**a**) FFE trend along longitude, and (**b**) FFE trend along latitude

**Fig. 10:**
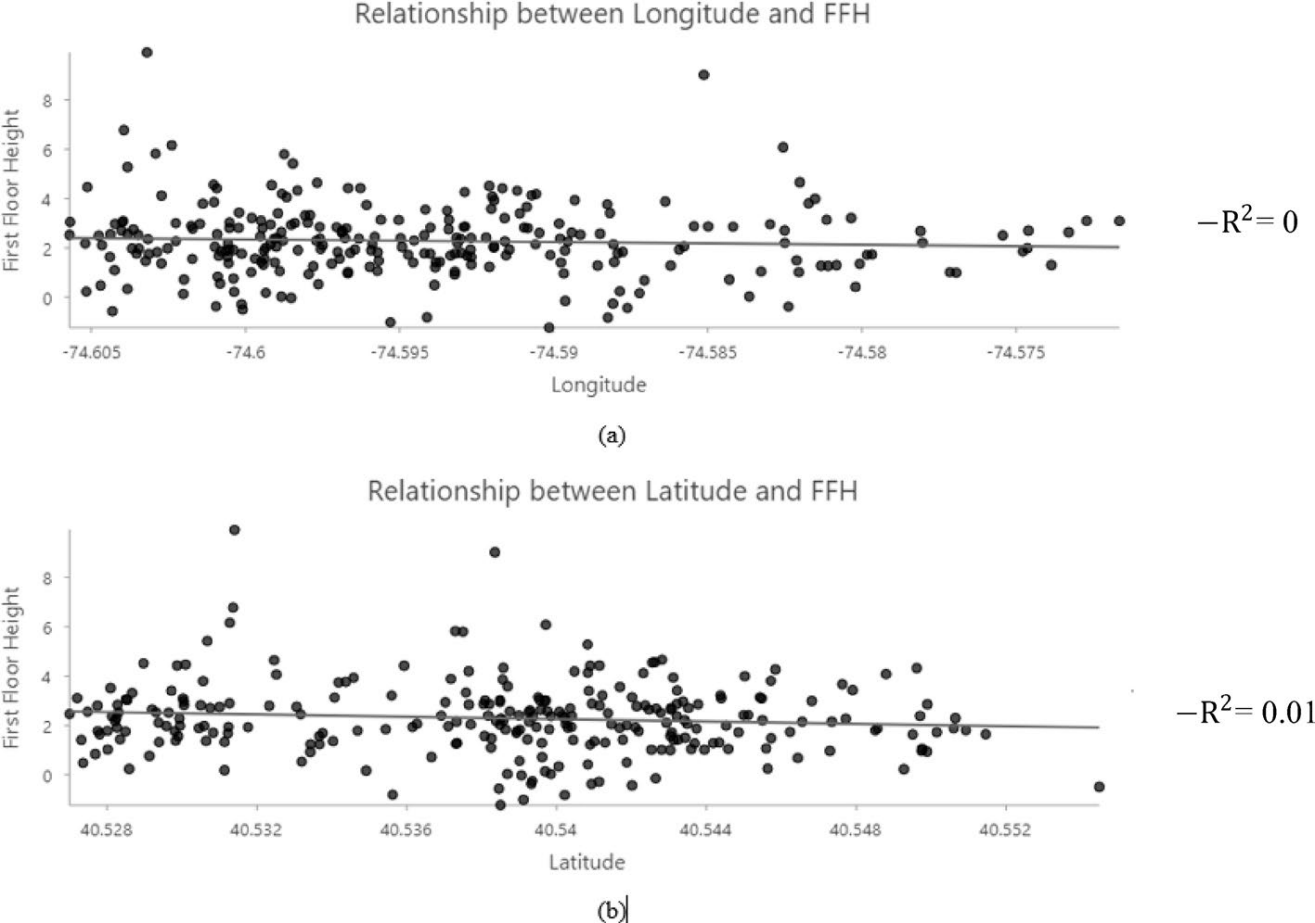
Relationship between First Floor Height (FFH) and geographic location: (**a**) FFH trend along longitude, and (**b**) FFH trend along latitude

Stationarity is also an important property for kriging, as kriging works best when the data’s statistical properties, such as mean and variance, remain constant. To assess the presence of large-scale spatial trends, scatter plots of First Floor Elevation (FFE) against longitude and latitude were examined (Fig. 9). The resulting R² values are used here as a descriptive diagnostic to indicate the strength of systematic spatial variation associated with geographic location, rather than as evidence of any causal relationship with ground elevation. Prior to any transformation, FFE exhibits a noticeable dependence on spatial location, with R² values of 0.37 and 0.14 for longitude and latitude, respectively. The magnitude of these R² values suggests that FFE contains a non-stationary mean component that is incompatible with the assumptions of ordinary kriging. To reduce this large-scale spatial trend and improve compatibility with the stationarity assumption, FFE was transformed into First Floor Height (FFH), defined as the elevation of the finished floor relative to the adjacent ground surface. After this transformation, the corresponding longitude–latitude plots (Fig. 10) exhibit near-zero R² values, indicating that the dominant large-scale trend associated with the topography has been removed while local spatial dependence that required for kriging interpolation remains.

**Fig. 11:**
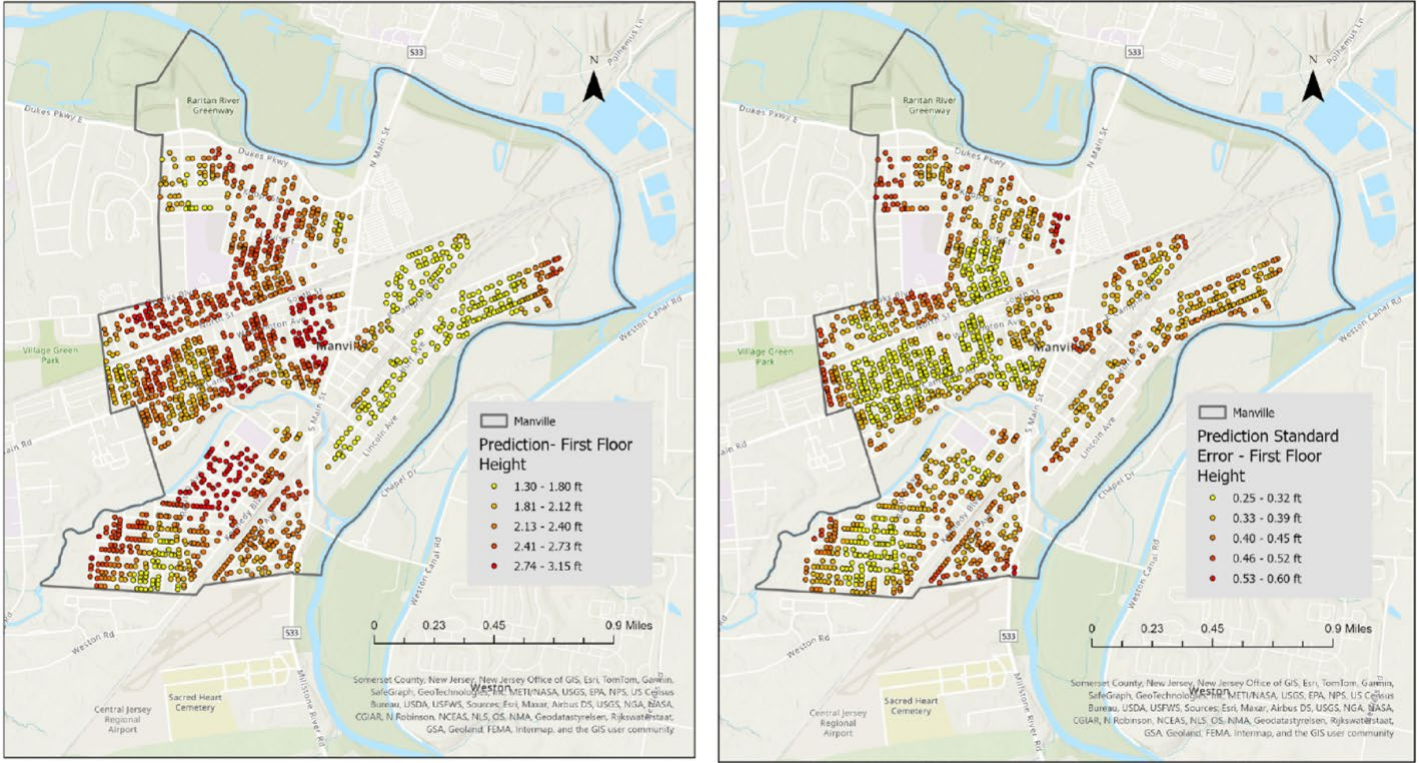
Kriging-based FFH prediction (left) and FFH Error Prediction (right)

The kriging analysis was conducted using ArcGIS Pro 3.1.0 Geostatistical Wizard for efficient spatial interpolation (ESRI, n.d.). This tool facilitated the development of variograms to model spatial dependency and applied ordinary kriging to predict First Floor Height (FFH) values. For illustration purposes, 20% of the data were used as a training set to predict the First Floor Height (FFH) for the remaining 80% of buildings. Figure 11 presents the kriging-based spatial prediction and corresponding error maps. The interpolated FFH surfaces were visualized using an equal-interval classification scheme based on the observed minimum and maximum predicted values within the study area to ensure consistent interpretation. Error magnitudes were grouped into standardized intervals. In this iteration, the model achieved a Root Mean Square Error (RMSE) of 0.9893 ft and a mean error of 0.0089 ft, indicating minimal systematic bias.

Following the validation procedure described in Sect.  3.4, model performance was evaluated using a repeated cross-validation strategy to ensure robustness to random subsampling. In each iteration, 20% of the available FFE observations were randomly selected as training data, and the remaining 80% were used for validation. This process was repeated five times using different random splits. Across the five cross-validation runs, RMSE values ranged from 0.969 to 1.129 ft, with a mean RMSE of 1.032 ft and a standard deviation of 0.06 ft, demonstrating stable model performance across different subsamples. Following commonly accepted survey and floodplain mapping accuracy standards—where Elevation Certificates and survey benchmarks often exhibit errors on the order of 0.5–1 ft and RMSE values within 1 ft are considered accurate, and 1–2 ft acceptable (Maune 1997)—the results for Manville indicate good predictive performance. Overall, the consistency of RMSE values across repeated subsamples confirms the reliability of the kriging-based approach for estimating First Floor Elevation in inland floodplain settings.

### Coastal town

The same validation framework applied in Manville was extended to two coastal towns, Longport and Ventnor City. In these coastal settings, buildings were classified into two categories—elevated and non-elevated based on the “elevated status” definition in Table 2. This simplified classification reflects both the prevalence of elevated construction as a flood-mitigation strategy in coastal areas and the limited sample sizes available for individual FEMA building-diagram categories, which precluded reliable diagram-level analysis.

In Longport, First Floor Elevation (FFE) values were successfully generated for 745 out of 1255 single family housing parcels. The remaining parcels were excluded due to limited façade visibility in the LiDAR point cloud caused by obstructions or unfavorable building orientation. Among buildings with valid FFE data, 7% (52) were classified as elevated and 93% (693) as non-elevated. In Ventnor City, FFEs were generated for 2838 out of 4252 single-family housing parcels, of which 23% (655) were elevated and 77% (2138) were non-elevated. Although elevated buildings represent a minority of the residential building stock in both towns, their presence reflects localized adaptation to coastal flood risk and motivates separate analysis.

**Fig. 12:**
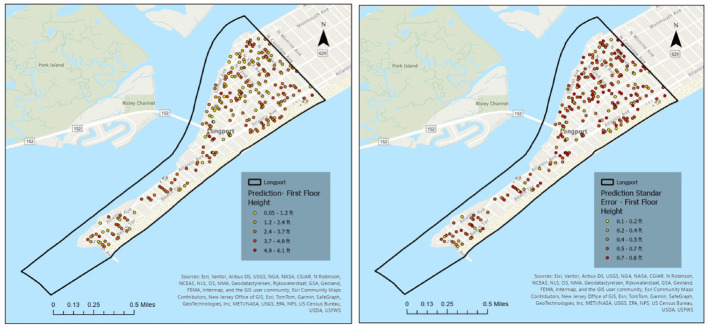
Kriging-based FFH prediction (left) and prediction standard error maps (right) for Longport Elevated Homes

**Fig. 13:**
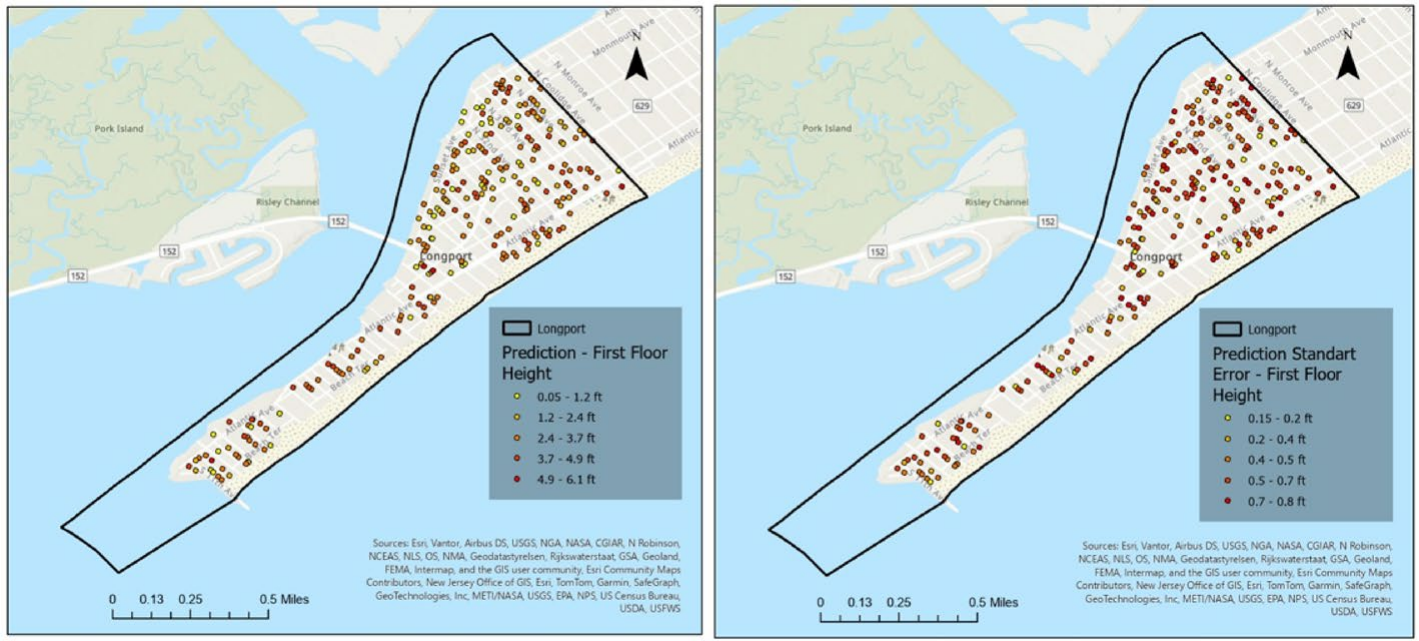
Kriging-based FFH prediction (left) and prediction standard error maps (right) for Longport Not Elevated Homes

To further support the spatial interpretation of model uncertainty, Figs. 12 and 13 present the kriging-based FFH prediction surfaces and corresponding prediction standard error maps for Longport, separated by elevated and non-elevated buildings. The spatial distribution of prediction error highlights localized clusters of higher uncertainty, particularly among elevated structures. These areas generally correspond to zones with sparsely observed FFE samples and greater variability in elevation practices. In contrast, non-elevated buildings exhibit more spatially homogeneous error patterns, consistent with their relatively uniform construction characteristics. The inclusion of these maps provides visual confirmation of the higher RMSE values reported for elevated buildings and supports the discussion of variability in coastal settings.

Following the validation procedure described in Sect.  3.4, repeated cross-validation was conducted using five random subsamples, with 20% of FFE observations used for training and the remaining 80% held out for validation in each iteration. In Longport, the mean RMSE for elevated buildings was 2.53 ft, while the mean RMSE for non-elevated buildings was 1.37 ft. The RMSE values across the fivefold cross-validation runs ranged from 2.43 to 2.64 ft for elevated buildings and 1.28–1.43 ft for non-elevated buildings, with corresponding standard deviations of 0.08 ft and 0.10 ft, respectively. The higher error observed for elevated structures indicates greater variability and reduced prediction precision compared to non-elevated buildings. This behavior is partly attributable to the aggregation of diverse elevated construction types into a single category, as well as to the unique coastal morphology of Longport and the prevalence of custom-built, high-value homes, which introduce additional heterogeneity in elevation practices.

Applying the same validation approach in Ventnor City, the mean RMSE values for elevated and non-elevated buildings were 1.64 ft and 1.41 ft, respectively. These error magnitudes fall within the expected and acceptable range for FFE estimation in coastal environments.The RMSE values resulted from the fivefold cross-validation procedure described in Sect.  3.4. In Ventnor City, repeated cross-validation produced RMSE values ranged from 1.57 to 1.71 ft for elevated buildings and from 1.17 to 1.95 ft for non-elevated buildings, with standard deviations of 0.07 ft and 0.47 ft, respectively, indicating stable performance across subsamples.

**Fig. 14:**
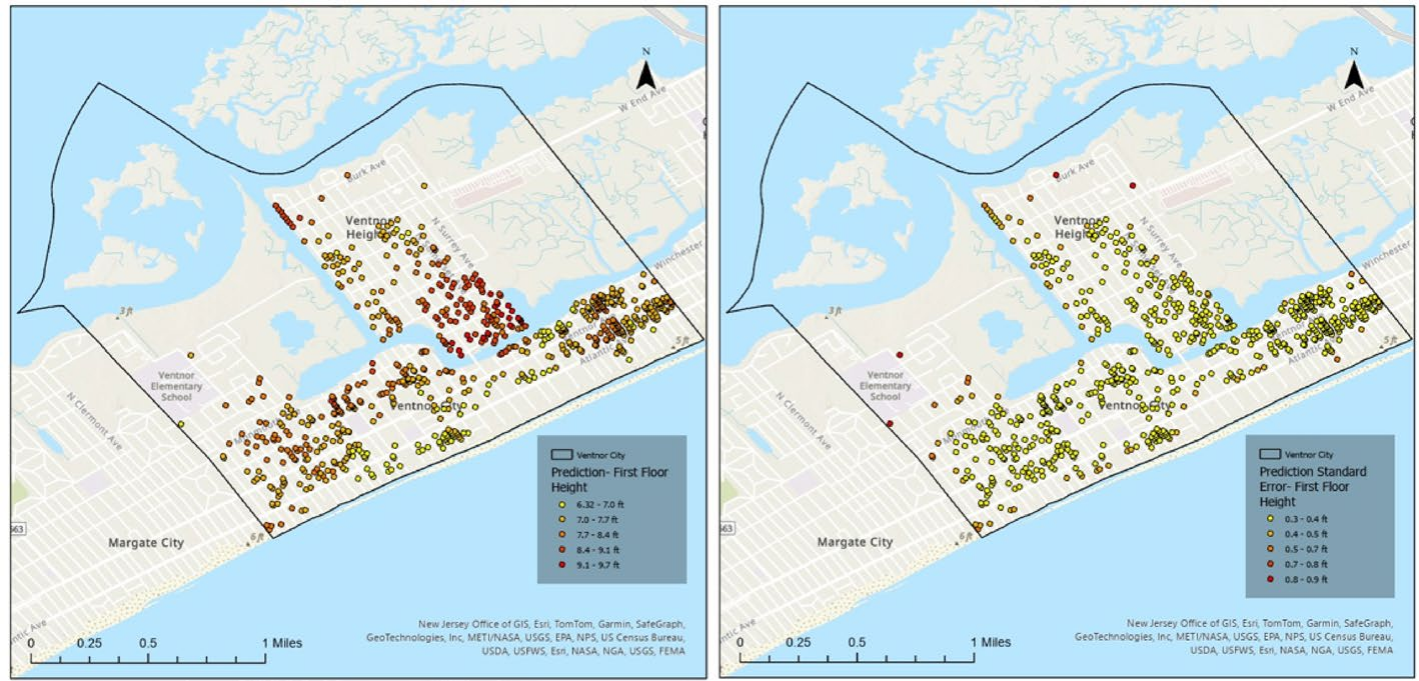
Kriging-based FFH prediction (left) and prediction standard error maps (right) for Ventnor City Elevated Homes

**Fig. 15:**
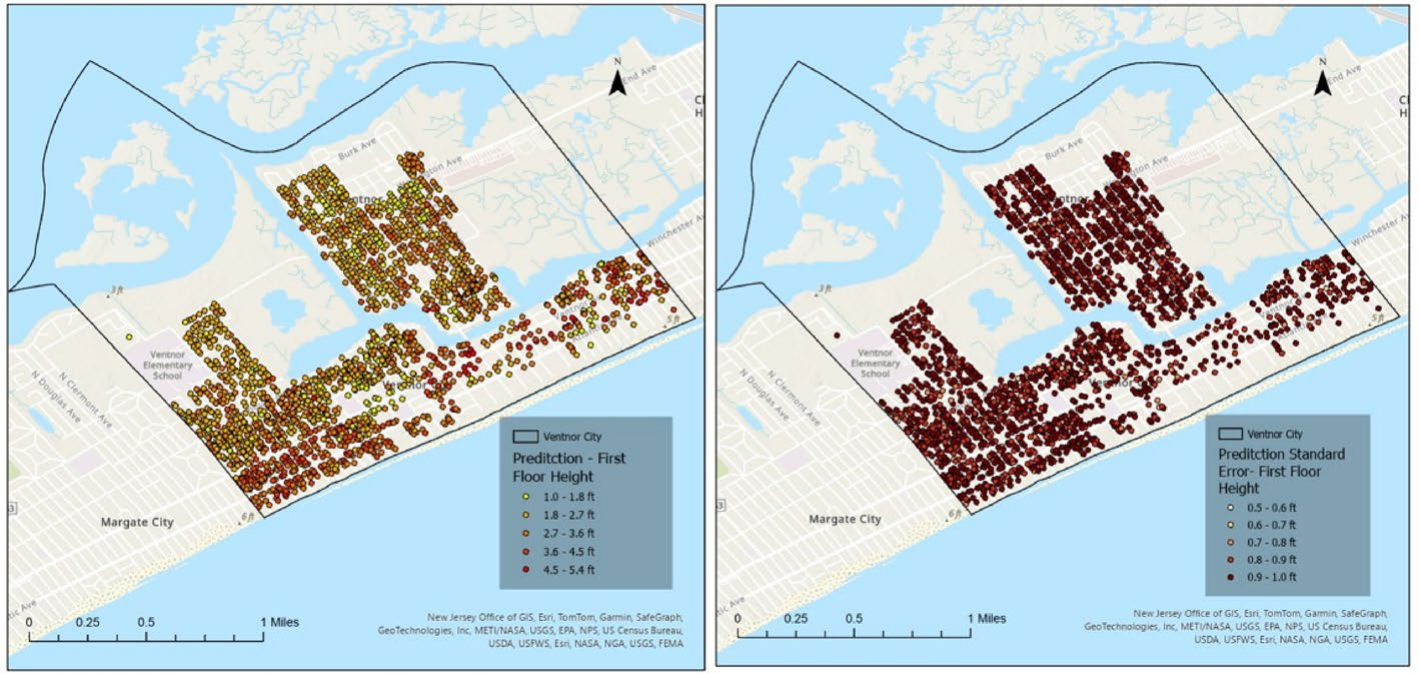
Kriging-based FFH prediction (left) and prediction standard error maps (right) for Ventnor City Not Elevated Homes

Figures 14 and 15 present the corresponding kriging-based FFH prediction and prediction standard error maps for Ventnor City, again separated by elevated and non-elevated buildings. Compared to Longport, Ventnor exhibits a more distributed spatial pattern of prediction uncertainty, reflecting its larger geographic extent and more heterogeneous building stock. Elevated structures display moderately higher localized error concentrations, while non-elevated buildings demonstrate relatively consistent prediction performance across the study area. These spatial error maps further substantiate the quantitative validation results and provide visual support for the discussion of coastal variability (see Discussion Sect.  5).

### Sensitivity analysis

**Fig. 16:**
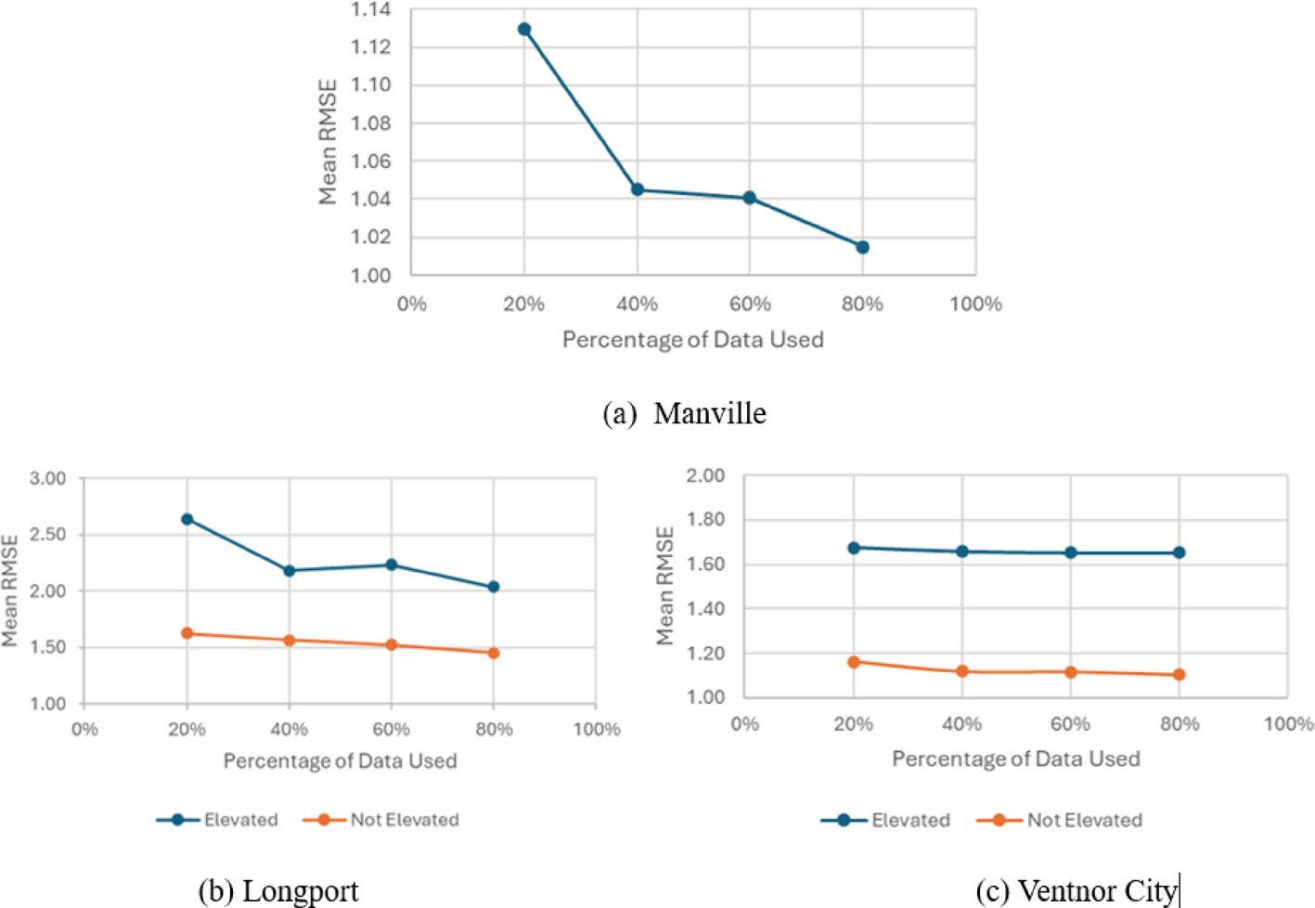
Changes in Mean RMSE (ft) with increased percentage of observed FFE, Manville (**a**), Longport (**b**), and Ventnor City (**c**)

To evaluate how predictive performance changes with increasing training data availability, training subsets corresponding to 20%, 40%, 60%, and 80% of the full dataset were randomly generated, with the remaining observations used for validation. For each proportion, this random sampling procedure was repeated five times, and the mean RMSE across the five runs is reported in Fig. 16 for the three townships. The RMSE at 20% training data in Fig. 16 does not exactly match the value reported in Sect.  4.2.2 and 4.3 because those results are based on a five-fold cross-validation procedure, whereas the sensitivity analysis relies on independently regenerated random subsets for each training proportion. The observed differences therefore reflect variation in data partitioning.

Overall, an increase in training data size improves model predictive accuracy across different townships. A larger sample improves the statistical reliability of predictions, contributing to reduced RMSE values. In particular, the performance after 40% remains relatively stable. This finding suggests that 40% elevation data is recommended for accurate prediction of FFE in a township. By expanding data collection efforts and including more diverse building types, future studies can refine the model’s applicability across varying urban landscapes and improve the precision of flood risk assessments on a broader scale. For coastal townships, the analysis revealed notable differences in accuracy between elevated and non-elevated buildings. Comparatively, non-elevated buildings show better prediction accuracy compared to elevated buildings. One of the main reasons is due to the freeboarding during the building elevation. Freeboarding serves to reduce flood risk, lower insurance premiums under the National Flood Insurance Program (NFIP) and enhance the long-term resilience of buildings. However, the discretionary nature of freeboarding creates challenges because homeowners may choose to exceed the mandated elevation requirements. This variation in elevation complicates efforts to establish consistent predictive parameters for elevated structures.

## Discussion

Three key findings are derived from the comparative analysis of inland and coastal townships. Collectively, they demonstrate how differences in building elevation practices, regulatory environments, and data composition influence the accuracy, variability, and generalizability of FFE prediction models.

The distinction between elevated and non-elevated buildings plays a critical role in determining the accuracy of FFE predictions. In the case study, the RMSEs of FFE prediction in coastal townships demonstrate that non-elevated buildings show better prediction accuracy compared to elevated buildings. Elevated buildings introduce higher variability in FFE due to differing construction practices and discretionary elevation heights, such as freeboarding, which are often applied inconsistently. In contrast, non-elevated buildings tend to follow more uniform construction patterns, leading to more stable and reliable predictions. As a result, grouping buildings into elevated and non-elevated categories captures the dominant elevation behavior effectively. Although more detailed classifications using FEMA building diagrams may provide improvements in prediction accuracy, dividing buildings by building elevation status is often enough in coastal areas where one type of building dominates or where there isn’t enough data to support more detailed classification.

Stronger local zoning and ordinance requirements related to building elevation are recommended for coastal regions, primarily to support flood-risk mitigation and long-term resilience. From a flood-management perspective, additional freeboarding, defined as voluntarily elevating structures above the mapped Base Flood Elevation remains a well-established and beneficial practice that reduces flood exposure and damage potential (“Freeboard” 2020). From a modeling perspective, however, elevated buildings in coastal areas exhibit higher prediction errors, as reflected by increased RMSE values, indicating greater variability in observed first-floor elevations relative to model expectations. This variability is likely influenced by discretionary elevation decisions, including freeboarding, as well as differences in local construction practices and permitting histories; however, the present analysis does not isolate or quantify the specific contribution of freeboarding to this uncertainty. Rather than framing freeboarding as a problem to be mitigated, these findings highlight a key challenge for data-driven elevation modeling: capturing real-world variability introduced by human decision-making. Improved access to detailed elevation records, such as typical freeboard practices, permitting data, and historical construction information, would allow future models to better represent this variability while preserving the flood-risk reduction benefits of elevated construction. In this context, clearer zoning guidance and more consistent documentation of elevation practices can enhance both regulatory transparency and model performance without discouraging adaptive flood-resilient design.

While expanding data collection to include a wider range of building types and urban contexts is essential for improving the generalizability and reliability of FFE predictions, increasing the sample size alone is not sufficient. Detailed building diagram classification, such as categorizing structures into 11 diagram types, still relies heavily on manual efforts, which can be time-consuming and resource-intensive. To reduce this burden, adopting a more generalized building classification system would be beneficial. Achieving this requires a systematic analysis of how specific building characteristics, such as foundation type and elevation status, influence FFE values. Furthermore, a comprehensive data collection strategy that integrates broader geographic sampling with key structural attributes is critical to enhancing the accuracy and robustness of geospatial flood risk models.

## Conclusion

First Floor Elevation (FFE) is a critical factor for building flood risk assessments. Approaches used in current FFE prediction studies often depend on detailed building data or high-quality images. In particular, statistical models need detailed building attributes like foundation type and year built. However, such data is often limited or incomplete because it is costly and time-consuming to collect. Image-based methods rely on aerial or street-view images, but their accuracy can be affected by poor image quality, blocked views from trees or buildings, and blurred features due to privacy rules. To address these questions, this research aims to design a systematic approach to predict FFE given limited FFE data. The proposed approach involves classifying buildings based on design attributes and then using Kriging to predict FFEs by leveraging spatial relationships and distances of buildings with FFEs.

Three New Jersey townships, Manville, Longport, and Ventnor City, were selected to validate the proposed methodology. The results highlight the importance of addressing data sparsity and leveraging spatial correlations to enhance flood risk modeling. With accurate and acceptable RMSEs, Kriging demonstrates its effectiveness as a robust method for estimating FFEs, particularly in regions where traditional survey-based data (e.g., elevation certificate) are sparse or unavailable. By leveraging spatial autocorrelation, the proposed approach fills a critical gap in existing literature that typically relies on large volumes of high-quality training data, thereby offering a solution for reliable FFE estimation in data-constrained regions. The case study also highlights the value of building classification (i.e., building diagram and elevation status) and spatial trends removal in the data (i.e., converting FFE to FFH) for accurately capturing spatial patterns in FFEs across buildings. The findings further emphasize the need for stronger local zoning and ordinance requirements for building elevation in coastal regions, a deeper understanding of how building characteristics influence FFE, and the development of a more generalized building classification system to support efficient and scalable FFE estimation.

The proposed approach can be applied in riverine or coastal areas with a modest set of Elevation certificates and building design attributes. While the methodology demonstrates strong generalizability, collecting detailed building design data for classification remains a labor-intensive process. Future research will focus on identifying the most influential building attributes affecting FFE through a sensitivity analysis. Based on these findings, a more generalized building classification framework will be developed to streamline data collection efforts for both riverine and coastal towns. This will enhance the efficiency, scalability, and practicality of FFE prediction models in diverse built environments.
